# Body weight and waist circumference are differentially associated with the response to L-thyroxine treatment in primary hypothyroidism

**DOI:** 10.1016/j.jcte.2026.100440

**Published:** 2026-04-18

**Authors:** Anders Funkquist, Stefan Sjöberg, Henrik Zetterberg, Stefan Bergman, Josefine Rosvall, Per Bjellerup, Johan Svensson

**Affiliations:** a Department of Medicine, Neurology, Halland County Hospital, Halmstad, Sweden; b School of Public Health and Community Medicine, Institute of Medicine, Sahlgrenska Academy, University of Gothenburg, Gothenburg, Sweden; c Department of Medicine, Karolinska Institute, Huddinge, Sweden; d Sophiahemmet University Stockholm, Sweden; e Department of Psychiatry and Neurochemistry, Institute of Neuroscience and Physiology, the Sahlgrenska Academy at the University of Gothenburg, Mölndal, Sweden; f Clinical Neurochemistry Laboratory, Sahlgrenska University Hospital, Mölndal, Sweden; g Department of Pathology and Laboratory Medicine, University of Wisconsin School of Medicine and Public Health, Madison, WI, USA; h Wisconsin Alzheimer’s Disease Research Center, University of Wisconsin School of Medicine and Public Health, University of Wisconsin-Madison, Madison, WI, USA; i Department of Neurodegenerative Disease, UCL Institute of Neurology, Queen Square, London, UK; j UK Dementia Research Institute at UCL, London, UK; k Hong Kong Center for Neurodegenerative Diseases, InnoHK, Hong Kong, China; l Centre for Brain Research, Indian Institute of Science, Bangalore, India; m Spenshult Research and Development Centre, Sweden; n Department of Pediatrics, Institute of Clinical Sciences, Sahlgrenska Academy, University of Gothenburg, Gothenburg, Sweden; o Department of Pediatrics, Halland Hospital Halmstad, Halmstad, Sweden; p Laboratory Medicine and Center for Clinical Research, Västerås, Sweden; q Department of Internal Medicine and Clinical Nutrition, Institute of Medicine, Sahlgrenska Academy, University of Gothenburg, Gothenburg, Sweden; r Region Västra Götaland, Department of Internal Medicine, Skaraborg Central Hospital, Skövde, Sweden

**Keywords:** Thyroid hormones, Orexin, (hypocretin), Metabolism, Hypothalamus, (L-thyroxine, Triiodothyronine), LDL-C, Cerebrospinal fluid

## Abstract

**Background:**

Thyroid hormones (TH) and neurotransmitter orexin (ORX) are implicated in the regulation of metabolism. Abdominal weight gain is common in primary hypothyroidism (PH).

**Methods:**

Our aim was to investigate whether TH affected peripheral weight gain, waist circumference (WC) and low-density lipoprotein cholesterol (LDL-C), before and 6 months after L-thyroxine substitution therapy. A secondary aim was to investigate the role of ORX.

**Results:**

Weight gain was positively correlated with improvement in QoL (r = 0.72, p = 0.003) and with CSF ORX levels in the 15 included patients (r = 0.78, p = 0.001). Increased WC, which was not associated with QoL changes, correlated negatively with free thyroxine levels, after 6 months of treatment, in both CSF (r = -0.71, p = 0.003) and serum (r = -0.64, p = 0.0097). Increased LDL-C correlated negatively with CSF free thyroxine levels after 6 months of treatment (r = -0.74, p = 0.003).

**Conclusion:**

The marked correlations with CSF levels of thyroxine and ORX suggest that hypothalamic mechanisms participate in the regulation of WC and weight during L-thyroxine therapy, highlighting the need for individualized treatment of the metabolic disturbances seen in PH.

**Short communication:**

Through evaluating the dynamics of body weight and waist circumference during the initial treatment of primary hypothyroidism, a positive correlation was found between orexin levels in CSF and the change in body weight. Furthermore, negative correlations were found between CSF free thyroxine levels and the changes in waist circumference and serum LDL-C levels. These findings emphasize the importance of finding future individualized treatment strategies in primary hypothyroidism, particularly regarding metabolic disturbances.

## Highlights

• Common misconception: body weight reflects metabolism; abdominal adiposity and cholesterol are more relevant markers.
• Primary hypothyroid patients were followed pre-treatment and after 6 months with metabolic, CSF TH, orexin, and QoL measures.
• TH regulate abdominal fat and cholesterol via ventromedial hypothalamic nc; CSF thyroxine correlated with waist and LDL after L-T4 initiation.
• TH system modulates orexin; weight change (not abdominal) correlated with baseline orexin and higher QoL after L-T4.
• Hypothalamic T4 regulates abdominal fat, while orexin regulates physical activity and muscle mass.

## Introduction

Primary hypothyroidism (PH) is an autoimmune disease characterized by reduced levels of thyroid hormones (TH), which negatively affect virtually all body organs and cellular functions. One of several prominent symptoms is weight gain [1]. Traditionally, body weight and the corresponding body mass index (BMI) have been used to assess the metabolic risk in humans; however, these measures do not capture changes in body composition. Referred studies have demonstrated that waist circumference (WC), reflecting visceral abdominal fat, is a stronger predictor of metabolic complications [2], [3]. In contrast, peripheral (not abdomen) weight gain, as opposed to gain in central obesity, may primarily reflect increased muscle mass and could therefore be associated with beneficial health outcomes [4]. Despite the well-established significance of metabolic alterations in PH, there is incomplete knowledge of the underlying mechanisms, especially in terms of the relations with TH levels in the central nervous system.

**Fig. 1:**
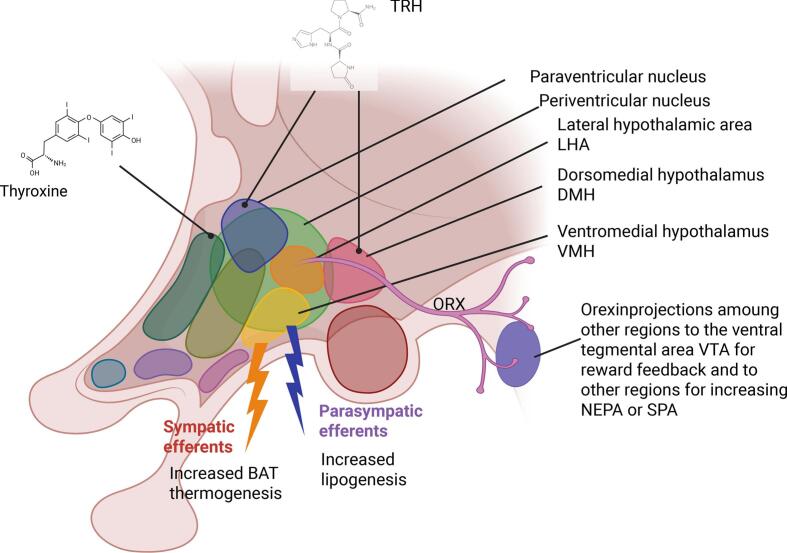
Illustration of the nuclei involved in metabolism in the hypothalamus. To keep the text more easily readable and simple the important nucleus Arcuate nucleus is not illustrated and not mentioned in this article. Figure created using BioRender.

TH, the active forms are mainly triiodothyronine (T3) and thyroxine (T4), which are essential regulators of metabolic homeostasis in mammals [5], [6]. Their primary mechanism of action involves binding to nuclear thyroid hormone receptors (TR), which in turn modulate the transcription of genes, predominantly those involved in anabolic processes. Beyond their direct impact on the peripheral targets such as muscle cells, TH plays a pivotal role in the complex networks regulating metabolism, growth, food-seeking behavior, and sleep. Specifically, TH influences the conversion of brown adipose tissue and the catabolism of fat in other tissues, a process mediated via the autonomic nervous system through the ventromedial hypothalamus (VMH) [7], [8], [9], [10] (Fig. 1). The influence of TH on the VMH occurs through the interaction with TR, specifically THRβ1, in the hypothalamus [6]. Given that T3 has a tenfold higher receptor affinity than T4, a major part of the metabolic effects is mediated by T3 [6]. In the brain, a metabolically highly sensitive organ, TH activity is tightly regulated by local conversion mechanisms. This regulation is enzymatically mediated by deiodinases (DIO) in astrocytes, which are strategically positioned near neurons to ensure precise metabolic control [11], [12]. Given the strict regulation on brain metabolism, the primary source of T3 in the VMH is from local conversion rather than direct peripheral supply [11], [12].

Orexin/hypocretin (ORX), a neurotransmitter identified in 1998 [13], plays a crucial role in sleep-awake regulation, food-seeking behavior, locomotor activity, and reward processing in the brain [13] (Fig. 1). ORX is synthesized by a small population of cells located in the lateral hypothalamic area (LHA). It modulates metabolic processes by affecting behavioral and mental patterns linked to arousal and foraging activity. Furthermore, ORX influences fundamental movement behaviors, commonly referred to as non-exercise physical activity (NEPA) or spontaneous physical activity (SPA). Experimental research suggests that ORX is at least partially regulated by neurons that use thyrotropin-releasing hormone (TRH) as their neurotransmitter [7], [14]. This TRH originates from the dorsomedial hypothalamus (DMH) [15], but its interactions with ORX are not fully understood. There exists, to date, no valid method for measurement of ORX in blood, but only in cerebrospinal fluid (CSF).

TH regulates cellular metabolism and as a consequence, metabolic disturbances, and alterations in body composition are often seen in states with low concentrations of TH as in patients with PH [16], [17]. Although recent research suggests that the metabolic effects of TH are partly mediated by hypothalamic nuclei [6], the effects of L-thyroxine replacement therapy on hypothalamic functions have been scarcely studied in humans. To be able to evaluate a hypothalamic impact from TH, values in CSF need to be measured.

The major aim of the present study was to determine whether TH, both in serum and CSF affected the relative distribution of peripheral weight gain, abdominal obesity and low-density lipoprotein cholesterol (LDL-C) before and 6 months after the start of L-thyroxine substitution therapy. A secondary aim was to investigate the role of ORX in relation to the metabolic parameters studied.

## Methods

### Study design

A cohort of patients with newly diagnosed PH, investigated before initiation of L-thyroxine therapy and followed up 6 months after the start of treatment.

### Study population

#### Flow diagram of patient follow-up

**Figure:**
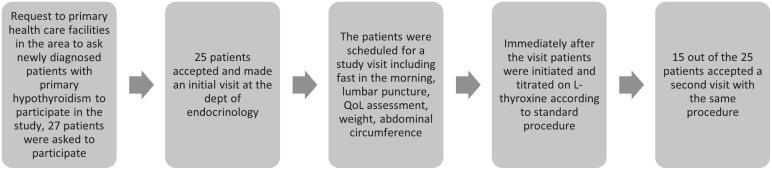

The main inclusion criteria was a newly diagnosed untreated PH, with an elevated TSH (n = 27). The patients were invited to the Unit of Endocrinology at the Department of Medicine, Halland Central Hospital Halmstad, Sweden. Due to the need for a homogenous population, we decided for only patients with an f-T4 level around the lower reference interval, and thus we needed also to validate their hypothyroid disease with the Zulewski scale. Two of the patients invited declined to participate. Ten patients did not attend a second visit due to: moving out of our region (n = 3); postpunctional headache or discomfort after the initial lumbar puncture (n = 4); pregnancy (n = 1); severe illness (n = 1); and being unreachable (n = 1). Exclusion criteria were treatment with anti-thyroid substances or thyroid hormones, recent use of iodine contrast, and pregnancy. Primarily, they were invited to the investigation by their primary care physicians at their primary health care centers. The recent onset of fatigue, hypersomnia, or lethargy were the main reasons for consulting their primary care physicians. Aside from PH, one subject was previously diagnosed with type-1 diabetes; four patients were treated with estrogen hormone replacement (oral estrogen or patch); and one subject received glucocorticoid inhalation. After these exclusions, 15 patients were eligible for the present study.

Data from 50 healthy controls, enrolled in a previous study [18], were used for reference comparisons in terms of absolute values, as the controls were only evaluated on one occasion. Besides this, the healthy controls were treated the same with all measurements and procedures as in the patients.

#### Initial visit

All patients displayed at least two pathologically increased serum TSH (>4.0 mIE/l; reference interval: 0.40–4.0 mIE/l) prior to admission and were evaluated according to the Zulewski scale [19], which ensured that they were clinically hypothyroid according to the suggestions from Zulewski et al. The patients were evaluated for any criteria of a depressive diagnosis based on medical record review and clinical psychiatric assessment at this first visit. Reference interval for f-T4 in serum was 11–22 pmol/L.

#### Second visit

The second visit took place after a median of 182 days (range: 119–623 days) following the first visit. A follow-up interval of approximately six months was chosen to minimize loss to follow-up. The clinical characteristics of the patients are presented in Table 1. All the 25 PH patients described previously [18], and included at the initial visit, were invited to a second visit, but due to dropout, 15 PH patients were included in the study. To ensure compliance, all patients had regular contact with the principal investigator during the entire study period. Furthermore, to ensure that TSH levels decreased in response to L-thyroxine treatment, blood sampling was done regularly during the study period. All participants were assessed by an experienced endocrinologist and received an appropriate dose of L-thyroxine in accordance with established clinical guidelines. Any dose adjustments, when indicated, were implemented between two and four months after baseline assessment, and all patients received a starting dose of 50 Âµg once daily. All patients reached s-TSH concentrations within the normal reference range. Descriptive data for the PH patients (n = 15, 11 women, mean (M) age: 52 ± 12 years) is presented in Table 1. All patients were of Caucasian origin, and none of them fulfilled any criteria for a depressive diagnosis based on medical record review and clinical psychiatric assessment at the first visit.

**Table 1:** Clinical characteristics in hypothyroid patients before and during thein mean 6 months of treatment, as well as in healthy controls. p-value for paired *t*-test between the two visits.

	**Before treatment** *M ± SD or* *n(percentage)*	**During treatment** *M ± SD or* *n(percentage)*	**P-value**	**Healthy controls***M ± SD or* *n(percentage)*
Gender (female/male)	11/4			25/27
Age (years)	52.0 ± 12.2			36.8 ± 11.4
Weight (kg)	84.4 ± 20.34	85.1 ± 20.6	0.25	75.4 ± 15.7
WC (cm)	99.0 ± 13.8	99.0 ± 16.9	0.99	86.5 ± 13.7
Time between visit one and two (days)		Md 182 min/max: 119–623		
s- TSH (mIU/l)	8.5 ± 9.7	0.8 ± 0.9	< 0.001	2.0 ± 1.2
s- f-T4 (pmol/l)	11.3 ± 2.6	17.1 ± 3.6	< 0.001	14.1 ± 1.9
CSF f-T4 (pmol/l)	8.5 ± 2.2	13.1 ± 2.5	< 0.001	9.6 ± 1.2
s- f-T3 (pmol/l)	5.4 ± 1.0	6.3 ± 1.3	0.05	6.2 ± 1.1
CSF f-T3 (pmol/l)	2.5 ± 0.9	2.0 ± 0.7	0.22	2.3 ± 0.6
s TPO-Ab > 15 IU/ml	10 (67%)	10 (67%)		6 (11%)
s TPO-Ab > 25 IU/ml	8 (53%)	9 (60%)		6 (11%)
Zulewski score ≥ 5	15/15	4/15	< 0.001	0
Dose of Levaxin® (µg)	0	92.5 ± 14.8		
Physical component summary of SF-36	44.23 ± 9.34	48.22 ± 9.38	0.11	55.88 ± 3.91
Mental component summary of SF-36	41.19 ± 9.94	47.00 ± 10.01	0.015	54.20 ± 4.91
CSF Orexin(ng/L)	655.4 ± 127.5	672.3 ± 142.9	0.22	672.3 ± 137.4
s- LDL-C (mmol/L)	3.32 ± 0.96	3.22 ± 1.03	0.76	2.83 ± 0.85

M = mean, SD = standard deviation, WC = waist circumference, s = serum, CSF = cerebrospinal fluid, TSH = thyroid-stimulating hormone, f-T4 = free thyroxine, f-T3 = free iodothyronine, TPO-Ab = thyroid peroxidase antibodies, LDL-C = low-density lipoprotein cholesterol.

### Body weight and waist circumference

Body weight was measured in the morning under fasting conditions of minimum 8 h, with a precision of 0.1  kg. The weight scale was calibrated yearly and was available in the hospital medical research unit. Waist circumference (WC) was measured in cm with the participant in a standing position, at the widest part of the abdomen using a standard clinical tape measure. Δweight and ΔWC were used to assess whether participants experienced gains or losses in overall body mass or adiposity (Δweight = weight in kg at second visit – weight in kg at initial visit, ΔWC = waist circumference at second visit – waist circumference at initial visit). Height was measured at each visit and remained stable throughout the study period. Therefore, any change in body weight directly corresponds to a proportional change in body mass index (BMI).

### Sampling of blood and CSF

All blood and CSF sampling was conducted after a fasting period of a minimum of eight hours, with collection taking place at 8 a.m. Venous blood samples were obtained via venipuncture and then centrifugated at 2400 x g for 10 min within 1–––3 h after collection and subsequently aliquoted into polypropylene tubes. The serum samples were stored at − 20 °C pending biochemical analyses, which included measurements of TSH, f-T3, f-T4, and thyroid peroxidase antibodies (TPO-Ab).

Lumbar puncture at the L4-5 intervertebral space was performed between 08:00–08:30 a.m. according to standardized procedures with the participants in a seated position. A disposable Quincke needle (0.70x75 mm, 22 GA; Becton-Dickinson, Oxford, UK) was used for the procedure. A total of 12  ml CSF was collected in polypropylene tubes and subsequently divided into six 2  ml of aliquots. The CSF samples were immediately transported to the local laboratory, centrifugated at 2400 x g in + 4 °C for 10 min, and then stored at − 70 °C until biochemical analysis. Samples had not been thawed and refrozen prior to the analysis.

### Biochemical procedures

The free components of TH were used rather than the total concentration as the unbound fraction is most relevant for the physiological effects and additionally, little is known for the binding to albumin and thyroglobulin in CSF. All serum and CSF samples were analyzed in the same assay run to minimize analytical inter-assay variation.

TSH, f-T3 and f-T4 were analyzed by dissociation-enhanced lanthanide fluorescence-immunoassays [20] (Auto DELFIA, Wallac Oy, Turku, Finland). The intra-assay coefficient of variation (CV) for the TSH assay was < 4.4% for all levels down to 0.1 mIE/l, and the intra-assay CVs for the f-T4 and f-T3 assays were < 6.1% for all levels down to 5 and 2 pmol/l, respectively.

TPO-Ab was analyzed by chemiluminescent micro particle immunoassay (CMIA) (Architect i2000, Abbott, Chicago, USA) [21]. The intra-assay CV was < 10% for all levels down to 5 IU/ml; >15  IU/ml was considered as a positive result.

LDL-C was calculated by Friedewald’s formula where HDL-cholesterol, total cholesterol and triglycerides were measured with standard enzymatic methods from Roche at the Modular system (Roche Diagnostics, Mannheim, Germany). Total CV was < 3% for all methods.

CSF orexin/hypocretin A1 (ORX) concentration was measured using a radioimmunoassay, as previously described [22]. The measurements were performed with the analyst blinded to clinical data. Total CV was < 6%. Because ORX levels exhibit diurnal variations, all CSF samples were collected between 08.00 and 08.30 a.m. following an overnight fast.

### Quality of life (QoL)

Following lumbar puncture and venous blood sampling, all participants performed a self-assessment of QoL using the Short Form-36 (SF-36) questionnaire [23]. From this, the physical component summary (PCS) and mental component summary (MCS) scores were calculated as previously described [24]. Z-scores was derived using normative coefficients based on Swedish population data [23], and the mental and physical component summary were calculated using the oblique factor scoring method described by Farivar et al. [24]. Higher scores indicate better perceived QoL.

All QoL assessments were conducted before, and on average six months after, the initiation of L-thyroxine treatment, allowing for the calculation of changes in QoL (post-treatment minus pre-treatment scores). Additionally, at the baseline visit, patients were asked whether they experienced fatigue (yes/no).

### Statistical methods

Statistical analyses were performed using R software (version 4.0.5, R foundation Vienna, Austria). If not otherwise stated, variables are presented as the mean ± standard deviation (M + SD). The assumption of normality was assessed using the Shapiro-Wilk test and QQ-plots. Due to deviations from normality with a left-skewed distribution, serum f-T4 levels during treatment were transformed using Box-Cox transformation [25]. In contrast, delta values of f-T4 in both serum and CSF and CSF f-T4 levels, which showed a right-skewed distribution, were log-transformed to achieve a normal distribution.

Within-group effects were evaluated by comparing pre- and post-treatment values using paired t-tests. Correlation analyses were conducted using Pearson’s correlation test, ensuring strict adherence to normality assumptions; variables that failed to meet these assumptions, even after an attempt for transformation, were excluded from the analysis. The categorical variable “fatigue at visit one” was coded as 0/1, and in the correlation test Pearson R correlation was used. A two-tailed p-value < 0.05 was considered statistically significant. To account for multiple comparisons of TH parameters, p-values were adjusted using the Holm method for seven different TH variables [26]. Furthermore, a multiple linear regression model was applied using the native lm function in R [27]. Potential covariates, for causal inference, were included in a Directed Acyclic Graph (DAG) model, using dagitty.net [28]. The potential covariates for all three regression models were initially all the other different measurements included in the study. The DAG formula was imported into R, and the dagitty::localTests function was applied to assess model assumptions, revealing which interaction variables could influence the model. This regression model was further utilized in the rms::fastbw() function, which iteratively removed variables with p-values exceeding 0.2. Additionally, the validate() function was applied to perform bootstrapping with 200 samples. The regression model for ΔWC was specified as follows: model <- lm(ΔWC ∼ log(ΔCSF f-T4 + Δweight + age before treatment). Given the sample size, and particularly the limited number of male participants, it was not feasible to include gender (as a categorical variable) in the DAG analyses or in the subsequent multiple regression models.

Since there is no existing data regarding TH in CSF and metabolic changes, a correct power analysis could not be done. It was possible to recruit 25 patients and 15 were available for the follow-up visit. It was still possible to apply the planned relevant statistical methods.

### Ethical considerations

The study was approved by the Regional Ethical Committee in Lund, Sweden (diary number 253/2008, 585/2009 and 491/2010). Oral and written informed consent were obtained from all participants.

## Results

### Changes in thyroid hormones, QoL and orexin

Clinical characteristics before and 6 months after the initiation of L-thyroxine treatment are presented in Table 1. Significant differences between visits were observed for TH parameters, where serum and CSF free thyroxine (f-T4) and serum free triiodothyronine (f-T3) but not CSF f-T3 levels were elevated after the start of treatment. Consequently, serum thyroid-stimulating hormone (TSH) levels were depressed. The SF-36 MCS and physical component score (PCS) measures were higher after treatment but were not reversed to the levels of healthy controls, as described in a previous report from the present study [29].

CSF ORX levels were similar in patients with PH and healthy controls; ORX levels were 655 ± 127 at visit one and 672 ± 143 at visit 2 in the PH patients compared with 672 ± 137 in the healthy controls (Table 1). The intra-individual variation in ORX levels between visits was minimal (16.9 ± 50.5).

### Change in waist circumference (ΔWC), and correlation to TH and ORX

There was no significant change in mean WC between visit one and two. However, the individual changes were highly variable as WC decreased in 6 patients and increased in 5 patients. Thus, WC was altered in 11 of the 15 PH patients.

There was no significant correlation between ΔWC and Δweight. Additionally, ΔWC did not correlate with CSF ORX levels at any of the visits, or with MCS and PCS at visit one, visit two, or the changes (Δvalues) in MCS and PCS. Furthermore, visit one WC showed no association with ΔWC.

A significant positive correlation was observed between ΔWC and the prescribed dose of L-thyroxine (r = 0.54, p = 0.038), meaning that a greater increase in WC was associated with a higher dose of L-thyroxine. The prescribed dose of L-thyroxine non-significantly tended to correlate negatively with the Zulewski score at visit two (r = -0.49, p = 0.062).

**Fig. 2:**
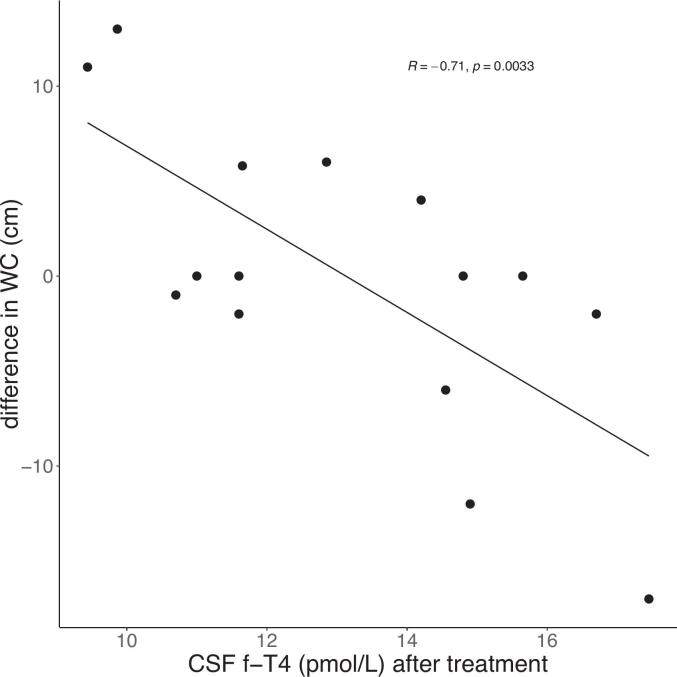
Pearson correlation for CSF f-T4 (cerebrospinal fluid concentration of free thyroxin) after in mean 6 months of treatment with L-thyroxin in patients with primary hypothyroidism and difference in waist circumference (ΔWC, (WC after treatment – WC before treatment)). N = 15, p = 0.0033.

Regarding TH levels, ΔWC exhibited significant negative correlations with CSF f-T4 (r = -0.71, p = 0.003) (Fig. 2) and serum f-T4 (r = -0.64, p = 0.0097) at visit two. Additionally, ΔWC was inversely correlated with the Zulewski score (r = -0.72, p = 0.003), Δserum f-T4 (r = -0.55, p = 0.034), and ΔCSF f-T4 (r = -0.63, p = 0.013).

There were no significant correlations between ΔWC and f-T3 values at visit one, visit two, or the changes (Δvalues) in f-T3.

To account for possible differences between sex the correlation between CSF f-T4 and ΔWC was analyzed separately for women and men. For women the results were significant and with about the same r-value (−0.68) and for men (n = 4) the r-value was −0.62 but not significant. To account for possible influence of menopause the correlation was calculated separately for women < 51 years old and above. Four women were at the age below 51 and had an r-value of −0.75 (n.s.) and 7 women was older than the average age for menopause in Sweden with an r-value of −0.68 (n.s.).

To account for potential confounding factors influencing the correlation between ΔWC and ΔCSF f-T4, a multiple linear regression model was applied with ΔWC as the dependent variable and CSF f-T4 at visit 2 as the independent variable, and an interaction term (CSF f-T4 at visit 2 x dose of L-thyroxine) as a covariate (Table 2). The covariates for all multiple linear regression models were decided with the help of a DAG analysis (see Methods). In the DAG analysis, Δweight, age at visit one and WC at visit one was also included but subsequently excluded in the following procedures in R. Due to the small sample size, the inclusion of additional variables was not feasible, but an additional test was performed including serum f-T4 at visit 2, which did not add any extra explanatory value, which was due to the high collinearity between serum and CSF f-T4 variables. The final regression model for ΔWC had an y-intercept of 25.77 and the independent variables β_1_(CSF f-T4 at visit 2) and β_2_(CSF f-T4 at visit 2 x dose of L-thyroxine) with an R^2^ of 0.69 (Table 2). This R^2^ was greater than the R^2^ derived from the Pearson correlation analysis above (R^2^ = 0.5), which suggests that CSF f-T4 levels at visit two together with the dose of L-thyroxine could explain almost 70% of ΔWC. An example for the interpretation of the algorithm is visualized in the appendix.

**Table 2:** β – coefficients including CI to be used in the 3 regression models.

		β-coefficient	p-value	Lower bound	Upper bound
	**β −coefficients for ΔWC regression model**				
β _1_	CSF f-T4 at visit 2	−3.50	0.0003	−5.0047	−1.9920
β _2_	CSF f-T4 at visit 2 x dose of L-thyroxine	+0.02	0.0182	0.0034	0.0300

	**β–coefficients for Δweight regression model**				
β _1_	ORX at visit 1	+0.02	0.0006	0.0085	0.0220
β _2_	CSF f-T4 at visit 2	+1.39	0.0354	0.1183	2.6576
β _3_	age at visit 1	−0.40	0.0221	−0.7209	−0.0714
β _4_	CSF f-T4 at visit 2 x weight at visit 1	−0.02	0.0193	−0.0313	−0.0036
β _5_	weight at visit 1 x age visit 1	+0.005	0.0189	0.0010	0.0083

	**β–coefficients for ΔLDL-C regression model**				
β _1_	CSF f-T4 at visit 2	−0.25	0.0023	−0.3851	−0.1091
β _2_	age at visit 1	−0.02	0.225	−0.0426	0.0112

ΔWC = changes in waist circumference, Δweight = changes in weight, ΔLDL-C = changes in LDL-C cholesterol.

### Change in body weight (Δweight), and correlation to TH and ORX

There was no significant change in mean weight between visit one and two in the PH group. However, weight changed between visits in all 15 patients, 7 patients with weight loss, and 8 patients with weight gain. As given above, no correlation was found between ΔWC and Δweight, and there was no association between weight at visit one (baseline) and the subsequent weight change (Δweight).

Δweight did not correlate with the dose of L-thyroxine (R = 0.39), TH levels in serum at visit one (f-T4: r = -0.20, f-T3: r = -0.31), visit two (f-T4: r = -0.16, f-T3: r = -0.41), or their respective changes (Δvalues) (f-T4: r = 0.06, f-T3: r = -0.15). TH levels in CSF at visit one (f-T4: r = -0.17, f-T3: r = -0.20), visit two (f-T4: r = -0.18, f-T3: r = -0.24), or their respective changes (Δvalues) (f-T4: r = -0.06, f-T3: r = -0.01). Similarly, no association was observed with the Zulewski scale at visit two (r = -0.20).

**Fig. 3:**
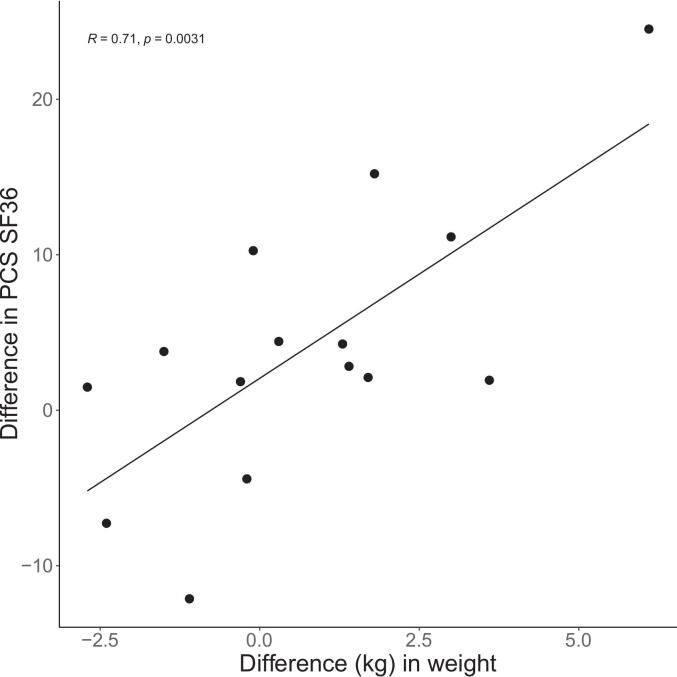
Pearson correlation between difference in weight (Δweight, (weight after in mean 6 months of treatment with L-thyroxin – weight before treatment)) and difference in physical component summary of SF36 (ΔPCS, (PCS after in mean 6 months of treatment – PCS before treatment)), in patients with primary hypothyroidism. N = 15, p = 0.0031.

Δweight showed significant positive correlations with the changes in the two SF-36 component scores: ΔPCS (r = 0.71, p = 0.003) and ΔMCS (r = 0.70, p = 0.003), indicating that gain in weight was associated with an improvement in these QoL assessments (Fig. 3). There were negative correlations between reported fatigue at visit one and Δweight (r = -0.52, p = 0.049) and reported fatigue at visit one and ΔPCS (r = -0.63, p = 0.011), indicating that a decrease in weight was correlated with a worsening of fatigue and physical health.

**Fig. 4:**
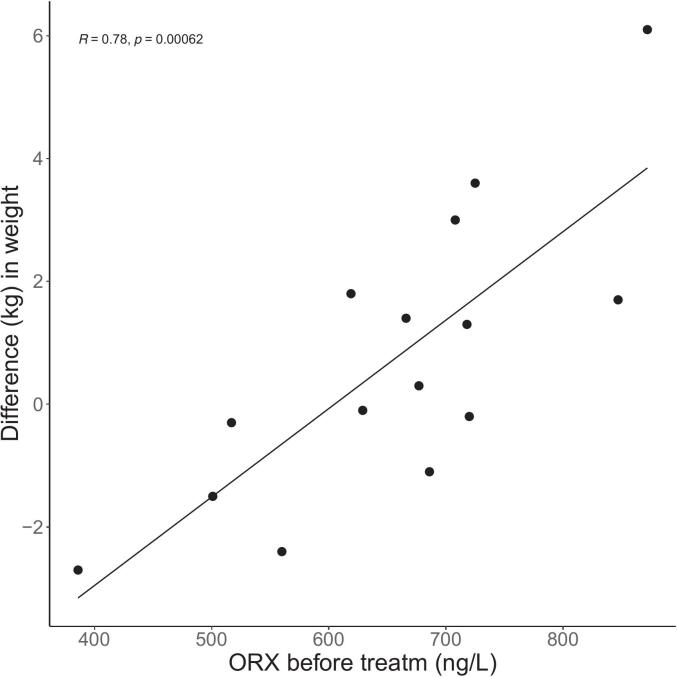
Pearson correlation between ORX level before treatment with L-thyroxin and difference in weight (Δweight, (weight after in mean 6 months of treatment – weight before treatment)), in patients with primary hypothyroidism. N = 15, p = 0.00062.

A positive correlation was found between Δweight and CSF ORX levels at visit one (r = 0.78, p = 0.001; Fig. 4) and visit two (r = 0.76, p = 0.001).

To assess potential confounding factors, a multiple linear regression model was applied, with Δweight as the dependent variable, ORX visit 1 as the independent variable and 2 interaction terms (CSF f-T4 at visit 2 x weight at visit 1) and (age at visit 1 x weight at visit 1) as covariates. Due to the small sample size, it was not feasible to incorporate additional variables, but in the initial DAG analysis ΔWC was included as a possible covariate and excluded in the following procedures. The final regression model for Δweight contained an y-intercept of −7.7 and the independent variables: β_1_(ORX at visit 1, β_2_(CSF f-T4 at visit 2), β_3_(age at visit 1), β4(CSF f-T4 at visit 2 x weight at visit 1), β5(weight at visit 1 x age visit 1)(Table 2). This model produced an R^2^ of 0.82. To obtain just one regression line from the multiple regression model (for comparison) all variables were set to their mean values except for ORX at visit one, whereby the resulting regression line was closely aligned with the line obtained by Pearson correlation.

The calculated R^2^ for the Pearson correlation between ORX at visit 1 and Δweight was 0.61, and adding the additional variables (CSF ORX at visit one, age, and CSF f-T4 at visit 2) in the multiple regression model gave an explanatory precision of 82% of the change in weight during treatment with L-thyroxine. An interpretation of the algorithm is visualized in the appendix.

### Changes in lipid profile, and correlation to TH and ORX

**Fig. 5:**
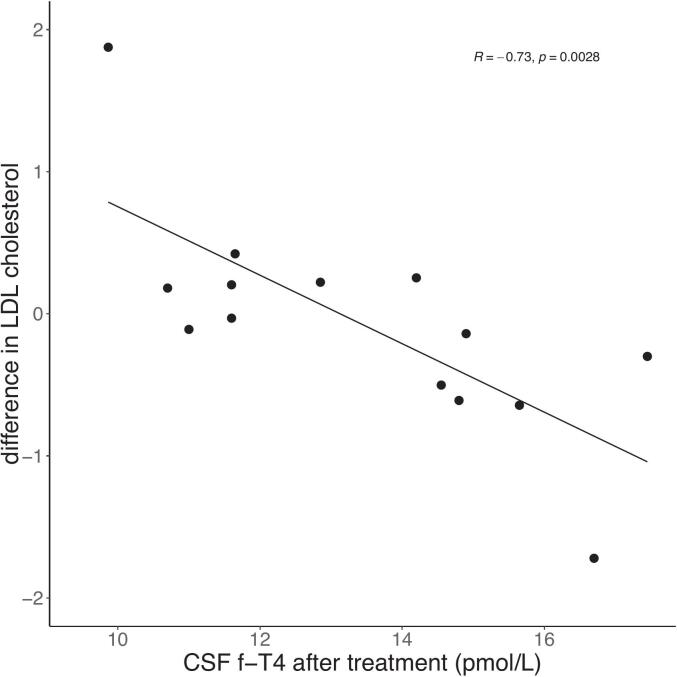
Pearson correlation between CSF f-T4 after in mean 6 months of treatment with L-thyroxin and difference in LDL-cholesterol (ΔLDL-C, (LDL-C after treatment – LDL-C before treatment)), in patients with primary hypothyroidism. N = 15, p = 0.0028.

Serum LDL-C levels were 3.32 ± 0.96 at visit one and 3.22 ± 1.03 at visit two. Given the observed correlation between f-T4 at visit two (both serum and CSF) and LDL-C, further analyses showed a significant negative correlation between ΔLDL-C and CSF f-T4 at visit two (r = -0.74, p = 0.003, Fig. 5). A similar, but weaker, negative correlation was observed between ΔLDL-C and serum f-T4 at visit two (r = -0.54, p = 0.047).

In a multiple linear regression model, using the same protocol as above, ΔLDL-C was included as the dependent variable; CSF f-T4 at visit 2 was used as the independent variable, and age at visit one was employed as a covariate. At first, the DAG analysis, which was used to find out covariates for multiple regression modelling, suggested that including the LDL-C value at visit one and an interaction term (age at visit 1 x LDL-C at visit 1), but this was excluded in the following procedures in R where certain p-value thresholds subsequently rules out covariates (see methods). Dose of L-thyroxine and WC at visit one was initially also included in the DAG analysis but were excluded in the following procedures. The final regression model contained an y-intercept of 4.06 and the independent variables β_1_(CSF f-T4 at visit 2) and β_2_(age at visit 1), which gave an R^2^ of 0.60. A model with serum f-T4 at visit 2 instead of CSF f-T4 at visit 2 was also constructed (the other covariate variable age the same), but this model produced an R^2^ = 0.42. To facilitate the interpretation of the algorithm, an example can be viewed in the appendix.

## Discussion

This study examined the associations between changes in body weight and WC in relation to CSF levels of TH and ORX in PH patients before and 6 months after the initiation of L-thyroxine treatment. There were no within-group differences in weight or WC between visit one and two, but the findings indicate that both weight and WC exhibit dynamic changes during L-thyroxine treatment regulated by distinct mechanisms. Weight variations were significantly associated with ORX levels at baseline and the same association with ORX at visit 2, whereas changes in WC and LDL were associated with CSF f-T4 levels at visit two and the difference between the both measurements (ΔCSF f-T4). The changes in WC and LDL-C were also correlated to serum f-T4 levels, but not as strongly as for CSF f-T4 levels. Overall, our results underscore a great variability in the response to L-thyroxine therapy and the importance of distinguishing between measurements of weight and WC. Furthermore, our results suggest that the availability of TH in CSF is important in the regulation of metabolic risk factors, particularly WC and LDL-C levels.

CSF levels can be used to estimate the levels of the analyte in the central nervous system (CNS). In the present study, the correlations between CSF f-T4 levels and the changes in WC and LDL-C were more marked than the corresponding correlations for serum f-T4 levels. Furthermore, in the multiple regression analyses, CSF T4 levels contributed to the models also after addition of covariates. Therefore, as T4 can be transported through the blood brain barrier, not only peripheral actions but also effects in the CNS induced by TH could be of importance for the effects on body composition and metabolism induced by L-thyroxine therapy, Furthermore, as discussed in more detail below, these findings combined with the strong correlations between CSF ORX levels and the changes in weight indicate that the CNS actions of L-thyroxine therapy may include effects on the hypothalamic ORZ system. The levels of ORX were like levels in healthy controls, but there was a strong correlation between the levels and weight change, hypothesized to be explained by the need for adequate levels of TH in the hypothalamus to exhibit a functional ORX system. The variation in ORX levels between the two visits was minimal, and the correlation with Δweight was the same, meaning that the participants did not exhibit any influence on ORX from possible changes in diet, physical activity or sleep habits.

Very few previous studies have investigated changes in CSF TH levels following the initiation of L-thyroxine treatment. In a previous study of pH patients, f-T4 but not f-T3 levels in CSF increased after the start of L-thyroxine treatment [30], which is consistent with the present findings. Apart from this study and our earlier investigation [29], TH concentrations in CSF following L-thyroxine treatment have previously not been reported. However, CSF levels of TH have been investigated in conditions such as Alzheimer's disease and other cognitive impairments. Although these studies may not be directly relevant for the current results, some but not all of the studies have found low CSF levels of T4 [31] or T3 [32] in Alzheimer’s disease.

### Waist circumference dynamics

Changes in WC (ΔWC) are associated with the risk of metabolic disease [33], [34]. Beyond the metabolic implications, an increase in WC is often perceived as more distressing by individuals compared to an overall increase in body weight as it more profoundly affects the shape of the body. In the present study, approximately one third of the PH patients experienced a reduction in WC, another third maintained stable WC, and the remaining third exhibited increased WC following six months of L-thyroxine therapy. Overall, on a group level, this means no change in WC. Furthermore, consistent with previous research [2], [35], ΔWC was not correlated with Δweight.

A central finding of the present study was that higher levels of f-T4 in CSF (and in serum) were associated with a reduction in WC. Conversely, a higher prescribed dose of L-thyroxine 2–4 months before visit two, was linked to lower f-T4 concentrations (in CSF and serum) at visit two, as well as a greater increase in WC, despite a reduction in serum TSH for all subjects. These findings highlight the complexity of TH metabolism and its impact on body composition, warranting further investigation in future studies.

Animal studies have demonstrated that T3 binds to receptors in the ventromedial hypothalamic nucleus (VMH), promoting lipid expenditure via the autonomic nervous system, which may in turn reduce WC [36]. For further insights, see the section “Effects of Thyroid Hormones on Metabolism”. Given that T3 has a higher turnover rate and a greater variability in concentration than T4, it is possible that effective lipid metabolism in the VMH depends on an adequate supply of T4 and sufficient DIO activity to convert T4 to T3 [12]. Supporting this hypothesis, patients with the highest CSF f-T4 levels at visit two exhibited the greatest reductions in serum LDL-C levels and WC.

Interestingly, physical symptoms of PH, as assessed by the Zulewski scale at visit two, were the lowest among the patients showing the greatest increase in WC. One might expect this group to have received the highest dose of L-thyroxine. However, there was a non-significant trend to a negative association between the dose of L-thyroxine and the Zulewski scale score at visit two. At the very least, this suggests that patients with the highest ΔWC had sufficient peripheral T4 concentrations, since they scored higher in the Zulewski score. However, the underlying mechanisms contributing to these findings require further investigation.

### Body weight dynamics

In the present study, which focused on individual metabolic changes, weight gain was associated with an improvement in self-reported QoL. The studied cohort exhibited ORX levels within the normal range. Notably, the highest ORX levels were observed in patients who had the most marked weight gain and the greatest improvements in the PCS and MCS subscores of the SF-36 health survey. Taking together, these findings suggest an interaction between the TH system and ORX, a hypothesis supported by several previous studies indicating that TRH modulates ORX activity [7], [14], [37].

Changes in body weight (Δweight) have been widely studied in the context of metabolic diseases. However, some of these studies have not included measurements of WC, which have been suggested as a more relevant indicator of metabolic health. Kahn et al. [2] highlighted that peripheral body weight and abdominal weight often exhibit contrasting trends, with the latter being more strongly associated with metabolic disease biomarkers. This may be supported by the results of a longitudinal study from Gothenburg, Sweden, which followed approximately 1,500 women over 24 years [38]. In this study, there was a negative correlation between BMI and mortality among individuals aged 50, 54 and 60 years at baseline, whereas a positive correlation was observed for waist-to hip ratio [38].

Relatively few studies have investigated the association between TH and body weight. In women with PH on L-thyroxine treatment, aerobic and/or resistance training improved lipid profile and physical health-related QoL [39]. However, the effects of this intervention on body weight or WC were not reported [39]. Another study on healthy individuals [40] identified a positive relationship between serum f-T4 (and f-T3) and weight loss, further supporting the role of TH in metabolic regulation. A previous study found that weight loss following treatment of pH was primarily due to excretion of excess body water rather than a reduction of fat mass [41]. A retrospective study from 2014 showed no significant weight loss after initiating substitution therapy [42] and pooled results from 2 individual studies on elderly with subclinical hypothyroidism did not show any group changes in weight or BMI [43]. In elderly patients with subclinical PH this was also shown in another study [44]. It is of relevance that the 2 participants in our present study that were older than 65 years (both were 70 years old) were showing among the lowest changes in both Δweight and ΔWC. Overall, there is a gap of knowledge regarding individual metabolic changes in relation to TH levels, particularly studies on central regulation of metabolism.

### Effects of orexin on metabolism

ORX was initially identified as an appetite-stimulating neurotransmitter [45]. However, subsequent research has demonstrated that ORX plays a broader role in metabolic regulation, acting as a protective factor against the development of metabolic syndrome and diabetes [46], [47], [48], [49]. The underlying mechanisms are likely multifaceted, involving both direct and indirect pathways.

A key metabolic function of ORX is its ability to stimulate brown adipose tissue (BAT) activity by enhancing sympathetic nervous system tone on this tissue [37], a physiological change that can take place also in human adults [50]. Additionally, ORX has been implicated in promoting non-exercise physical activity (NEPA) or spontaneous physical activity (SPA), although the mechanisms underlying this effect remain unclear. In the present study, PH patients appeared to exhibit ORX levels within the normal range. However, it seems like the L-thyroxine substitution treatment was required for ORX to exert its regulatory effects on NEPA. Given the well-established relationship between TRH and ORX producing neurons [7], [9], [14], it is plausible that alterations in TRH levels during treatment influenced the functional impact of ORX.

The appetite-stimulating properties of ORX are primarily mediated via the braińs reward system, with significant projections to the ventral tegmental area (VTA), a crucial dopamine center involved in motivation and reinforcement behaviors [51] (Fig. 1). This suggests that ORX plays a complex role in energy homeostasis, integrating metabolic needs with behavioral responses to food availability and activity. Given the strong association between the ORX system and reward mechanisms, the observed improvements in QoL parameters in this study may to some extent be attributable to interactions between the reward system and ORX activity, where the latter seems to be dependent on adequate TH levels in the involved hypothalamic nuclei.

### Effects of thyroid hormones on metabolism

A comprehensive review of the metabolic actions of TH by Sinha et al. [6] as well as another review [10] highlighted the critical role of the VMH in metabolic regulation (Fig. 1). While multiple factors, including glucose and leptin, contribute to this regulation, TH (particularly T3) plays a pivotal role. The metabolic effects of TH are mediated through two TH receptors: TH binding to TRα induces lipogenesis via increased parasympathetic activity while simultaneously activating BAT through enhanced sympathetic stimulation, which stimulates this tissue to mitochondrial lipid oxidation [52], and TH binding to TRβ results in decreased food intake and an increased locomotor activity [36].

In the present study, CSF f-T4 concentrations were significantly associated with the changes in WC (ΔWC) and serum LDL-C (ΔLDL-C). Given that TH, particularly T3, plays a crucial role in BAT activation and lipid metabolism, CSF f-T4 probably serves as a primary TH source for the VMH, where it could facilitate these metabolic processes.

### Strengths of the study

There are very few studies—regardless of the disease being examined—that have performed two lumbar punctures on the same individuals to track individual changes over time. The present study followed patients from before the initiation of L-thyroxine treatment until after 6 months of treatment, allowing for an individualized assessment of metabolic changes in PH. This longitudinal design combined with the availability of CSF biomarkers before and after the initiation of treatment is a setup that previously has only been explored in animal studies, which have shown similar results as the present study [36], [48], [53], [54].

Moreover, all biochemical analyses were conducted in high-quality laboratories using reproducible and state-of-the-art methods, ensuring the reliability and validity of our results.

### Limitations

Our study primarily focused on metabolism in relation to CSF values of TH and ORX. Although additional data on food habits and physical activity would have been of interest, self-reported anamnestic data on food intake are often subject to biases, including recall bias and the potential influence of an individual’s current quality of life status. A physical capacity test, for example a bicycle ergometer test or handgrip as for muscle mass, could have been of interest in this study.

Additionally, our sample size consisted of only fifteen PH patients. A larger sample would therefore have been preferable to improve the statistical power as well as the generalizability of our findings. However, conducting two lumbar punctures in the same individual is a challenging procedure, making large-scale studies difficult. A six-month follow-up could be regarded as too short but was chosen due to the risk of loss to follow up.

### Conclusions

This study on metabolic changes, measured as variations in weight, WC and LDL-C, demonstrates that individual changes in these biomarkers are strongly associated with ORX levels for weight dynamics and with thyroid hormones changes, predominantly in CSF, for changes in WC (ΔWC) and serum LDL-C (ΔLDL-C).

#### Practical implications

This article presents two key findings:

• Weight change and orexin: the change in weight observed over six months of initial L-thyroxine treatment for primary hypothyroidism (PH) is related to ORX, often referred to as the “activity hormone”. Additionally, this weight change is linked to improvements in quality of life (QoL).
• Waist circumference (WC) and f-T4: the change in WC is negatively associated with the concentrations of f-T4 in CSF and to some extent also with serum f-T4. Notably, this change in WC is independent of QoL changes and weight variations.

These findings suggest that intrinsic hypothalamic changes play a crucial role in the disease course. Our most important take-home message is that there appear to be complex physiological changes in the balance of TH in different hypothalamic nuclei and activity of different DIO that L-thyroxine therapy alone cannot probably fully address.

## CRediT authorship contribution statement

**Anders Funkquist:** Writing – original draft, Visualization, Validation, Project administration, Methodology, Investigation, Formal analysis, Data curation, Conceptualization. **Stefan Sjöberg:** Writing – review & editing, Supervision, Project administration, Investigation, Funding acquisition, Conceptualization. **Henrik Zetterberg:** Writing – review & editing, Formal analysis. **Stefan Bergman:** Writing – review & editing, Supervision. **Josefine Rosvall:** Writing – review & editing. **Per Bjellerup:** Writing – review & editing, Formal analysis. **Johan Svensson:** Writing – review & editing, Supervision, Conceptualization.

## Declaration of competing interest

The authors declare that they have no known competing financial interests or personal relationships that could have appeared to influence the work reported in this paper.

## Appendix A Supplementary data

Supplementary Data 1 Examples of interpretation of the multiple regression analysis.
