# Supplementary material for: Body weight and waist circumference are differentially associated with the response to L-thyroxine treatment in primary hypothyroidism
Source: J Clin Transl Endocrinol. 2026 Apr 18;44:100440. doi: 10.1016/j.jcte.2026.100440 (PMC13137142; doi:10.1016/j.jcte.2026.100440)
Supplement: Supplementary Data 1 — Examples of interpretation of the multiple regression analysis. [file mmc1.docx]

**Supplementary equations**

Examples of interpretation of the multiple regression analysis

For the dynamics in waist circumference (WC), the results of the multiple regression analyses indicated that a patient with a CSF f-T4 value at visit 2 of 17 pmol/L and a prescribed dose of L-thyroxine of 75 µg has a 70 % chance of lowering WC with: 25.77 - (3.5×17) + (0.02 x (17x75)) = -8.23 cm.

For the dynamics in weight, the results of the multiple regression analyses suggested that a patient with a CSF orexin (ORX) value at visit 1 of 850 ng/L, age of 45 years, weight at visit 1 of 85 kg, and CSF f-T4 at visit 2 of 17 pmol/L has an 80 % chance of a weight gain of: -7.7 + (0.02×850) + (1.39×17) – (0.40×45) – (0.02 × 17 × 85) + (0.005 × 85 × 45) = 5.2 kg. In contrast, a CSF ORX value at visit 1 value of 400 ng/L, with all other variables held constant, is associated with an 80% probability of a weight change of -3.8 kg.

For the dynamics in LDL-C values, a patient with a CSF f-T4 value at visit two of 10 pmol/L and age of 45 years has a 62% chance of having a change in LDL of: 4.06 – (0.25 × 10) – (0.02 × 45) = 0.7 mmol/L while a patient with CSF f-T4 value at visit two of 18 pmol/L and age of 45 years has a 62% chance of LDL-C change: 4.06 – (0.25 × 18) – (0.02 × 45) = -1.3 mmol/L.
